# Cytoplasmic polyadenylation and cytoplasmic polyadenylation element-dependent mRNA regulation are involved in *Xenopus *retinal axon development

**DOI:** 10.1186/1749-8104-4-8

**Published:** 2009-03-02

**Authors:** Andrew C Lin, Chin Lik Tan, Chien-Ling Lin, Laure Strochlic, Yi-Shuian Huang, Joel D Richter, Christine E Holt

**Affiliations:** 1 Department of Physiology, Development and Neuroscience, University of Cambridge, Downing Street, Cambridge, CB2 3DY, UK; 2 Program in Molecular Medicine, University of Massachusetts Medical School, Plantation St, Worcester, MA 01605, USA; 3 Department of Physiology, Anatomy and Genetics, University of Oxford, Parks Road, Oxford, OX1 3PT, UK; 4 Cambridge Centre for Brain Repair, Department of Clinical Neurosciences, University of Cambridge, Robinson Way, Cambridge, CB2 2PY, UK; 5 Institut National de la Santé et de la Recherche Médicale, Biologie des Jonctions Neuromusculaires, Université Paris V, Paris, France; 6 Institute of Biomedical Sciences, Academia Sinica, 128 Sec. 2 Academia Road, Taipei 11529, Taiwan

## Abstract

**Background:**

Translation in axons is required for growth cone chemotropic responses to many guidance cues. Although locally synthesized proteins are beginning to be identified, how specific mRNAs are selected for translation remains unclear. Control of poly(A) tail length by cytoplasmic polyadenylation element (CPE) binding protein 1 (CPEB1) is a conserved mechanism for mRNA-specific translational regulation that could be involved in regulating translation in axons.

**Results:**

We show that cytoplasmic polyadenylation is required in *Xenopus *retinal ganglion cell (RGC) growth cones for translation-dependent, but not translation-independent, chemotropic responses *in vitro*, and that inhibition of CPE binding through dominant-negative interference severely reduces axon outgrowth *in vivo*. CPEB1 mRNA transcripts are present at low levels in RGCs but, surprisingly, CPEB1 protein was not detected in eye or brain tissue, and CPEB1 loss-of-function does not affect chemotropic responses or pathfinding *in vivo*. UV cross-linking experiments suggest that CPE-binding proteins other than CPEB1 in the retina regulate retinal axon development.

**Conclusion:**

These results indicate that cytoplasmic polyadenylation and CPE-mediated translational regulation are involved in retinal axon development, but that CPEB1 may not be the key regulator of polyadenylation in the developing retina.

## Background

The assembly of functional neural circuits in the developing nervous system requires axonal growth cones to respond appropriately to guidance cues to lead axons to their correct targets [1]. Growth cone chemotropic responses to many guidance cues require local axonal translation and induce global translation activation [2-5]. However, axons are estimated to contain approximately 100–200 mRNAs [6,7], and guidance cues do not induce the translation of all of them. Indeed, guidance cues that have different effects on growth cones induce translation of different proteins, such as β-actin or CREB (cAMP response element binding protein) for some attractive cues [8-10] versus RhoA or cofilin for some repulsive cues [4,11]. RNA-binding proteins regulating axonal mRNAs are starting to be identified [8,9,12,13] but, overall, the mechanisms underlying mRNA-specific regulation of local axonal translation remain unclear.

Control of poly(A) tail length is an attractive candidate mechanism for mRNA-specific regulation of axonal translation. With a few exceptions (for example, core histones), the efficiency of translation of an mRNA depends on the length of its poly(A) tail. Poly(A) binding protein (PABP), together with the cap binding factor eukaryotic initiation factor 4E (eIF4E), helps recruit eIF4G, which indirectly binds the 40S ribosomal subunit to the 5' end of the mRNA, thereby stimulating initiation [14-17]. Specific sequence elements in some mRNAs recruit RNA-binding proteins that control poly(A) tail length, allowing mRNA-specific translational regulation by cytoplasmic polyadenylation.

The most well-understood mechanism for controlling cytoplasmic polyadenylation is regulation of mRNAs containing the cytoplasmic polyadenylation element (CPE; consensus UUUUUAU) by CPE-binding protein (CPEB)1. According to current models [18], CPEB1 binds to CPE-containing mRNA and associates with a large complex of proteins, including the scaffolding protein Symplekin [19] and cleavage and polyadenylation specificity factor (CPSF) [20,21]. CPEB1 also binds to a poly(A)-specific ribonuclease (PARN) and the atypical poly(A) polymerase Gld2 (Germ-line development factor 2), which are responsible for deadenylating and polyadenylating CPEB1 target mRNAs, respectively [19,22]. PARN is the more active enzyme, so when both PARN and Gld2 are present, the poly(A) tail remains short and the mRNA is silenced [22]. Phosphorylation of CPEB1 by the kinase Eg2/Aurora A [23] induces the dissociation of PARN from the CPEB1-containing complex, allowing Gld2 to elongate the poly(A) tail [22], leading to polyadenylation-induced translational activation. Other RNA-binding proteins, such as Musashi [24] and Nanos and Pumilio [25], have also been implicated in regulating cytoplasmic polyadenylation, but their mechanisms of action remain unknown.

Although CPEB1 was first discovered in the maturation of *Xenopus *oocytes [26], it has since been implicated in diverse functions ranging from cell cycle control [27] to cell senescence [28]. In addition, CPEB1 regulates local translation in dendrites [18], a system that, like axonal translation, allows distal outposts of the neuron to respond quasi-autonomously to local stimuli [5]. CPEB1 regulates dendritic localization, polyadenylation, and translation of CPE-containing mRNAs such as CaMKIIα (Ca^2+^-calmodulin-dependent protein kinase II α) [29,30]. N-methyl D-aspartate (NMDA) receptor signaling induces both phosphorylation of CPEB1 via Aurora A kinase and polyadenylation of CaMKII mRNA at synapses [31], and induces translation of CPE-containing mRNAs in a polyadenylation-dependent manner [32]. CPEB1 knockout mice have subtle defects in learning and memory, showing reduced fear extinction [33] and in certain forms of long-term potentiation [34]. Overexpression of dominant-negative CPEB1 can cause defects in cerebellar long-term depression and motor learning [35] or dendritic arborization [36].

The function of cytoplasmic polyadenylation in diverse systems from *Xenopus *oocyte maturation to synaptic plasticity suggested that this might be a conserved mechanism for translational regulation in neurons and, therefore, a good candidate for regulating translation in axonal growth cones. Therefore, we asked whether cytoplasmic polyadenylation and CPEB1 might play a role in regulating translation for growth cone chemotropic responses. We found that translation-dependent, but not translation-independent, growth cone chemotropic responses require cytoplasmic polyadenylation. CPEB1 protein, however, is not detected in the retina and CPEB1 loss-of-function does not cause retinal axon guidance defects. UV cross-linking experiments show that other CPE-binding proteins are present in the retina and, indeed, dominant-negative inhibition of CPE-binding causes defects in axon outgrowth. Together, these results suggest that both cytoplasmic polyadenylation and CPE-mediated translational regulation are important for retinal ganglion cell (RGC) axon growth and guidance.

## Results

### Inhibition of polyadenylation blocks Semaphorin3A-induced growth cone collapse

Bath application of Semaphorin3A (Sema3A) causes *Xenopus *RGC growth cones to collapse, that is, to lose their filopodia and lamellipodia and assume a thin, non-motile form [37]. Sema3A-induced growth cone collapse occurs maximally at 10 minutes and requires local protein synthesis [2,11]. To address whether Sema3A-induced collapse requires cytoplasmic polyadenylation, we used the polyadenylation inhibitor 3'deoxyadenosine (cordycepin). When converted to cordycepin 5'triphosphate (3'dATP), it inhibits polyadenylation [38] by acting as a chain terminator due to the lack of a 3' hydroxyl group. Cordycepin, which does not affect protein kinase activity, inhibits cytoplasmic polyadenylation and meiotic maturation in *Xenopus *oocytes [39,40] and CPE-mediated translational activation in hippocampal neurons [32]. We incubated cultures with 200 μM cordycepin for 30 minutes to allow the cordycepin to enter the growth cone and be converted to cordycepin triphosphate to be added to poly(A) tails as a chain terminator, and then treated the cultures with Sema3A for 10 minutes.

**Figure 1:**
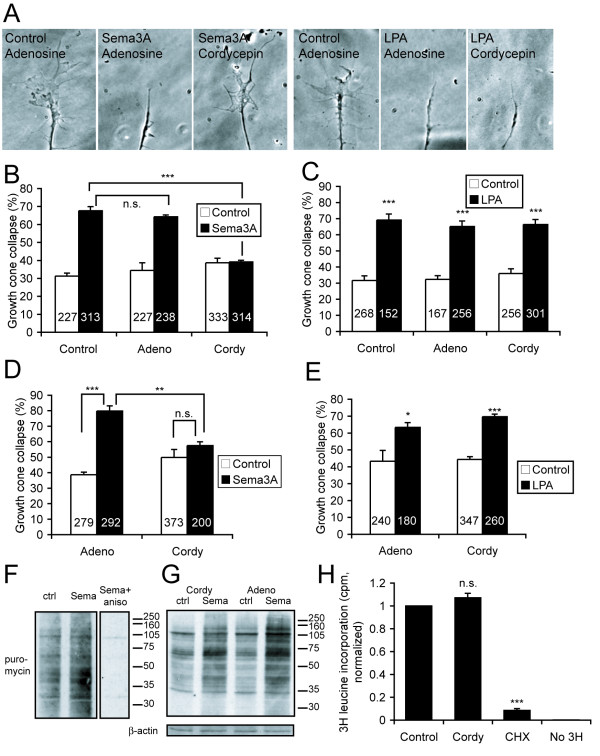
**Cytoplasmic polyadenylation is required for growth cone collapse**. **(A) **Typical examples of collapsed and non-collapsed growth cones. **(B) **Cordycepin (200 μM; 3'deoxyadenosine; Cordy), but not adenosine (Adeno), blocks Sema3A-induced growth cone collapse. **(C) **Cordycepin does not affect lysophosphatidic acid (LPA)-induced growth cone collapse. **(D, E) **Similar results are obtained as in (B, C) if axons are severed from their cell bodies. **(F, G) **Anti-puromycin western blots on stage 35/36 retinal cultures incubated for 15 minutes with 2 μM puromycin and 50 μM LnLL (F) or 0.5 μM puromycin (G), ± 2 μg/ml Sema3A-Fc, ± 40 μM anisomycin (aniso), ± 200 μM cordycepin or adenosine. Cordycepin slightly reduces, but does not abolish, Sema3A-induced translation (G); see text for details. The two separate sections in (F) are from the same blot with the same film exposure and contrast settings. **(H) **Cordycepin does not block basal translation in A6 fibroblasts, while cycloheximide (CHX) does. Numbers in bars indicate number of growth cones counted. *p < 0.05, **p < 0.01, ***p < 0.001. Error bars represent standard error of the mean.

We found that cordycepin, but not adenosine, completely abolished Sema3A-induced growth cone collapse (Figure 1A, B). In contrast, cordycepin had no effect on growth cone collapse in response to lysophosphatidic acid, another repulsive cue that does not require protein synthesis for its effects [2] (Figure 1C). This result indicates that cordycepin does not have non-specific toxic effects on growth cone responsiveness or collapsing capability.

To rule out effects of cordycepin on the cell body, we severed axons from their cell bodies before treating them with cordycepin and Sema3A. Again, cordycepin blocked Sema3A-induced collapse but not lysophosphatidic acid-induced collapse (Figure 1D, E). This result implies that cytoplasmic, not nuclear, polyadenylation is required for collapse.

We used two methods to exclude the possibility that cordycepin abolishes translation of all mRNAs. First, we labeled newly synthesized proteins in Sema3A-stimulated and control retinal cultures with puromycin, a chain-terminating tRNA analogue that tags the carboxyl terminus of nascent proteins [41,42]. At the concentrations used in this study (0.5–2 μM), puromycin can label all nascent proteins, both full-length and incomplete, which produces an indistinct 'smear' of puromycin labeling when labeled proteins are separated by SDS-PAGE and detected by anti-puromycin western blot (Figure 1F). Puromycin labeling is abolished by the peptidyl transferase inhibitor anisomycin (Figure 1F). Note that the distinct bands (as opposed to the 'smear') in Figure 1F, G are from non-specific binding by the anti-puromycin antibody, because the same bands also appear on samples incubated with the peptidyl transferase inhibitor anisomycin (Figure 1F) and on samples not incubated with puromycin (data not shown). Sema3A stimulation causes an increase in puromycin incorporation; this increase is slightly reduced, but not abolished, by cordycepin (Figure 1F, G). Because puromycin labels the mixture of full-length and incomplete proteins, this slight reduction in puromycin incorporation could represent either a reduction in overall protein synthesis or the blockade of synthesis of specific proteins.

Second, we tested the effect of cordycepin on basal translation rates in A6 cells, a *Xenopus *kidney cell line. We incubated A6 cells with ^3^H-leucine for 5 minutes and measured the incorporation of ^3^H-leucine into trichloroacetic acid-insoluble material by scintillation counting. Cordycepin pre-treatment had no effect on incorporation of ^3^H-leucine, while the protein synthesis inhibitor cycloheximide almost completely abolished it (Figure 1H). Together with the puromycin experiment, these results suggest that cordycepin is not a general translation inhibitor under these conditions and, thus, most likely exerts its effects through blocking polyadenylation.

### CPEB1 mRNA is expressed at low levels in the embryonic retina

Given that cytoplasmic polyadenylation is required for growth cone collapse, we considered the mechanisms by which cytoplasmic polyadenylation could be regulated in growth cones. CPEB1 was a good candidate for playing a central role in this process for several reasons. First, CPEB1 regulates translation via cytoplasmic polyadenylation in several systems, from *Xenopus *oocytes to both mammalian and invertebrate neurons. Second, CPEB1 is by far the most well-characterized regulator of cytoplasmic polyadenylation and has been especially well-studied in *Xenopus*. Finally, according to large-scale *in situ *hybridization studies, the mouse and zebrafish homologs of CPEB1 (known as *zorba *in zebrafish) are expressed in the embryonic retina [43,44].

**Figure 2:**
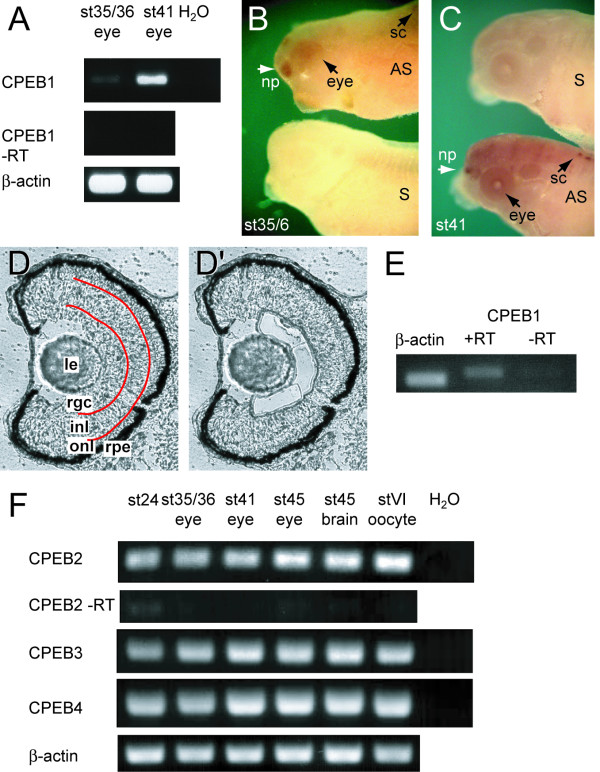
**CPEB1 mRNA is expressed in the *Xenopus *embryonic retina**. **(A) **RT-PCR shows that CPEB1 is expressed in stage 35/36 and stage 41 eyes and that the level of expression is higher at stage 41. **(B, C) **Wholemount *in situ *hybridization on stage 35/36 (B) and stage 41 (C) embryos shows that CPEB1 is weakly expressed in the eyes, as well as the nasal placode and brain, and in a segmented pattern in the dorsal spinal cord. The sense probe gives no signal. AS, antisense; np, nasal placode; S, sense; sc, spinal cord. **(D) **Section of stage 41 eye before (D) and after (D') laser capture microdissection of the retinal ganglion cell layer. Abbreviations: inl, inner nuclear layer; le, lens; onl, outer nuclear layer; rgc, retinal ganglion cell layer; rpe, retinal pigment epithelium. **(E) **RT-PCR on RNA from the retinal ganglion cell layer isolated by laser capture microdissection shows that CPEB1 is expressed in retinal ganglion cells. **(F) **CPEB2–4 are also expressed in embryonic eyes and brains by RT-PCR.

Given the conserved role of CPEB1 in regulating translation via cytoplasmic polyadenylation in systems ranging from *Xenopus *oocytes to mammalian and invertebrate neurons, we asked whether CPEB1 is expressed in *Xenopus *RGCs. We found by RT-PCR and wholemount *in situ *hybridization that CPEB1 mRNA is expressed in *Xenopus *stage 35/36 and stage 41 eyes (Figure 2A–C). CPEB1 is also expressed in the nasal placodes and in a segmented pattern in the dorsal spinal cord and weakly in the brain (Figure 2B, C; data not shown). The first pioneer RGC axons grow through the optic tract at stage 35/36, arriving in the tectum at stage 37/38 and arborizing at stage 39 [45], but because RGCs are born from stage 25 through stage 33/34–35/36 [46-48], many later-born RGC axons are still navigating through the optic tract at stage 41. Thus, CPEB1 is expressed at the right time and place to play a role in axon guidance. It should be noted, however, that the CPEB1 signal detected by *in situ *hybridization in the eye and brain is weak when compared to the robust signal in other areas such as the nasal placodes and spinal cord (Figure 2B, C). To address whether CPEB1 is present in RGCs, *in situ *stained wholemount embryos (Figure 2B, C) were sectioned but the *in situ *signal was too faint to be clearly seen in the thin tissue, although the RGC layer did not exhibit an obvious lack of signal relative to the other layers (data not shown). Therefore, to confirm that CPEB1 is expressed specifically in RGCs, we used laser capture microdissection to extract RNA specifically from the RGC layer of stage 41 eyes (Figure 2D), using this stage because the retina is not fully laminated at stage 35/36. This technique excludes other retinal layers, although we cannot completely exclude the presence of mRNAs from non-RGC neuroepithelial or glial cells whose endfeet reside in the RGC layer. RT-PCR showed that CPEB1 mRNA is present in stage 41 RGCs (Figure 2E).

The other members of the CPEB family, CPEB2–4, are also expressed in stage 35/36 and stage 41 eyes (Figure 2F), but because there is no evidence that these proteins control cytoplasmic polyadenylation [49], we focused our attention on CPEB1.

### CPEB1 loss-of-function does not cause defects in retinal axon pathfinding

**Figure 3:**
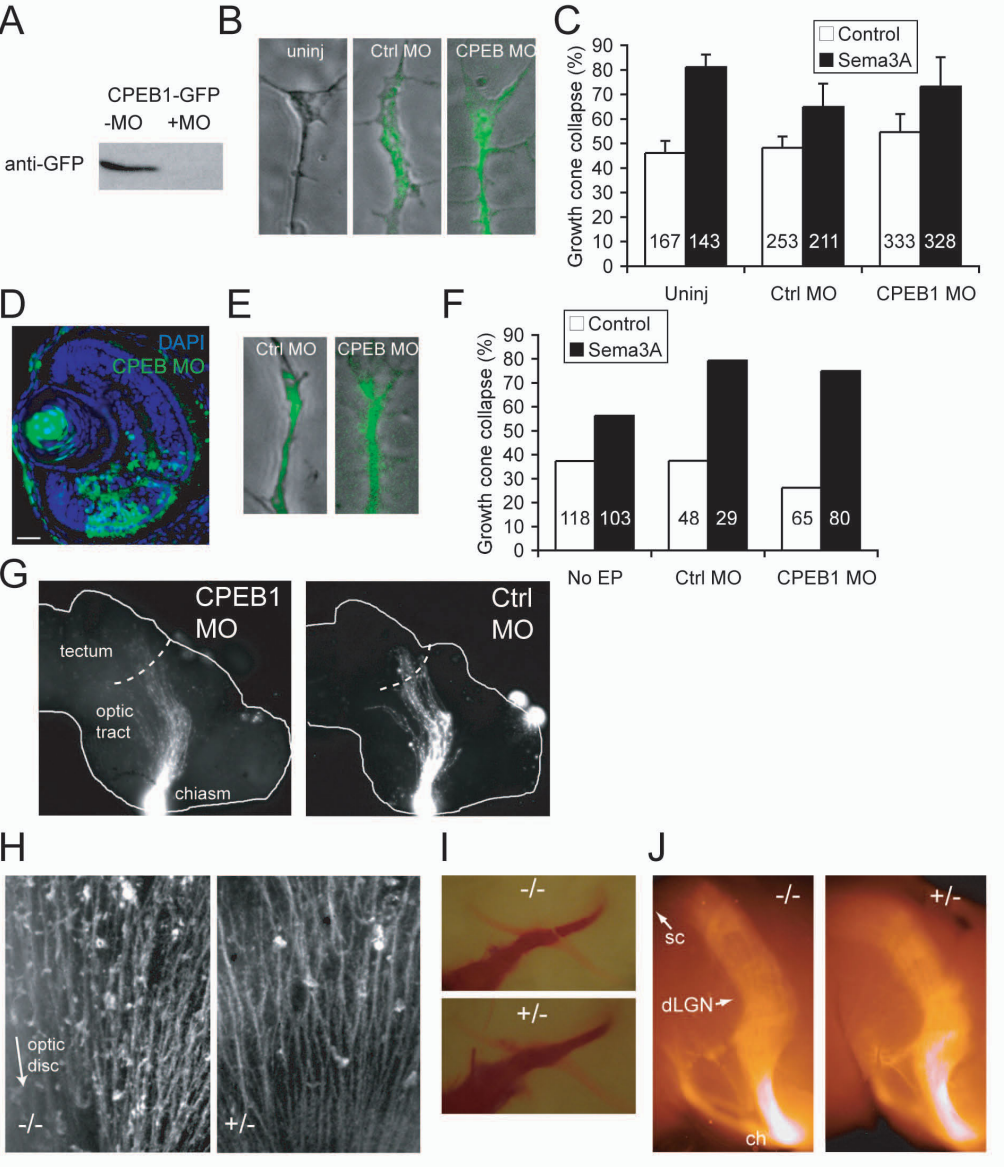
**CPEB1 loss-of-function does not cause obvious retinal axon guidance defects**. **(A) **Anti-green fluorescent protein (GFP) western blot on lysates of stage 24 embryos injected with 1 ng CPEB1-RBM-GFP mRNA with or without CPEB1 morpholino (MO) at the two-cell stage. CPEB1 MO effectively knocks down expression of CPEB1-GFP. **(B) **DiI filling of the retinal projection of stage 41 *Xenopus *embryos injected with CPEB1 MO at the two-cell stage shows no obvious defect in *in vivo *axon pathfinding. Uninj, uninjected. **(C-G) **Blastomere injection (C-D) and electroporation (EP) (E-G) of CPEB1 MO does not affect Sema3A-induced growth cone collapse. Blastomere injection (C) and electroporation (E, F) of carboxyfluorescein-tagged CPEB1 MO labels cell bodies (E) and growth cones (C, G) containing the MO with green fluorescence. **(H) **Beta-tubulin staining of wholemount mouse retinas shows no obvious defect in intraretinal guidance in embryonic day (E)18 CPEB1 knockout mice; retinal axons converge normally on the optic disc. **(I, J) **DiI filling of the retinal projection at E18–19 reveals no obvious defect in retinal axon guidance at the optic chiasm (F) or in the optic tract (G). Abbreviations: ch, optic chiasm; dLGN, dorsal lateral geniculate nucleus; sc, superior colliculus. Error bars represent standard error of the mean.

We next asked whether knocking down CPEB1 function would affect growth cone chemotropic responses and axon guidance, using antisense morpholino oligonucleotides, which effectively and specifically block translation of target mRNAs [50,51]. A carboxyfluorescein-tagged antisense morpholino (MO) directed against the ATG start site of CPEB1 mRNA successfully blocked translation of CPEB1-RBM-GFP mRNA (CPEB1-RNA binding mutant -green fluorescent protein; see below) when 20 ng MO and 2 ng mRNA per embryo were co-injected into blastomeres at the two-cell stage (Figure 3A). Retinal axons cultured from injected embryos contained carboxyfluorescein-tagged MO (Figure 3B). Surprisingly, blastomere injection of CPEB1 MO did not affect Sema3A-induced growth cone collapse (Figure 3C). To avoid the possibility that the CPEB1 MO may not be effective 80 hours after injection [50], we electroporated the CPEB1 MO into the eye at stage 28 (32 hours post-fertilization), which labeled retinal cell bodies (Figure 3D) and growth cones (Figure 3E). Again, the CPEB1 MO did not affect Sema3A-induced growth cone collapse (Figure 3E). Consistent with this, blastomere injection of 20 ng CPEB1 MO per embryo had no obvious effect on axon pathfinding through stage 41 *in vivo*, as assessed by DiI labeling (Figure 3F).

Similarly, the CPEB1 knockout mouse [52] does not have obvious retinal axon guidance defects. We assessed intraretinal guidance by staining wholemount retinas for beta-tubulin, and overall retinal axon pathfinding by labeling RGC axons with DiI, in embryonic day (E)17–E19 mouse embryos. No obvious differences were seen between CPEB1 knockout mice and heterozygous littermates (Figure 3H, F). Consistent with this, aside from subtle defects in learning and memory, the CPEB1 knockout mouse does not have obvious behavioral, motor, or sensory defects [33,34], as might be expected if axon pathfinding were perturbed.

### *Xenopus *embryonic retinas express multiple CPE-binding proteins

**Figure 4:**
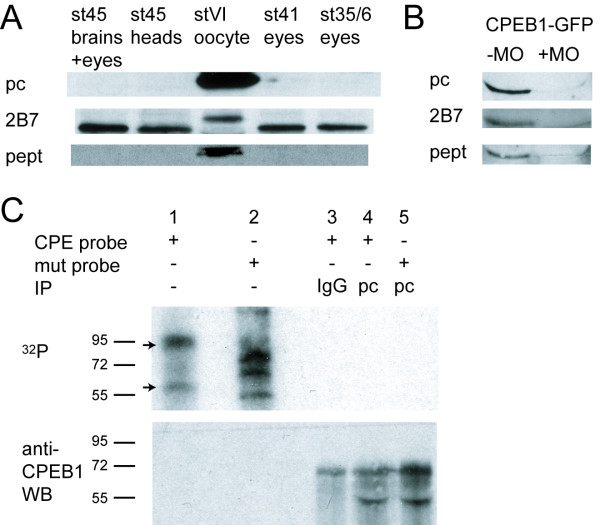
***Xenopus *embryonic retinas contain non-CPEB1 cytoplasmic polyadenylation element (CPE)-binding proteins**. **(A) **Western blots using three different antibodies do not detect CPEB1 protein in stage 45 eyes, stage 45 heads, stage 41 eyes, or stage 35/6 eyes. However, they do detect CPEB1 in stage VI oocytes. pc, polyclonal antibody against amino terminus of *Xenopus *CPEB1; 2B7, monoclonal antibody against human CPEB1; pept, polyclonal antibody against amino-terminal peptide of *Xenopus *CPEB1 (see Materials and methods). **(B) **All three antibodies used detect recombinant CPEB1 in stage 24 embryos injected with CPEB1-RBM-GFP mRNA (Figure 3A). MO, morpholino. **(C) **UV cross-linking with ^32^P-labeled CPE-containing probe suggests the presence of non-CPEB1 CPE-binding proteins. Stage 41 eye extracts were incubated with ^32^P-UTP labeled probe consisting of the 3' end of *Xenopus *cyclin B1 with CPE motifs intact (lane 1) or mutated (lane 2), and resolved by SDS-PAGE. Two bands are labeled with ^32^P (upper panel; indicated by arrows) but not detected by anti-CPEB1 western blot (lower panel). Proteins binding only to the mutated probe (lane 2) may recognize sequences masked by the CPE-binding proteins in the intact CPE-containing probe. Cross-linked samples were also subject to immunoprecipitation (IP) by control IgG (lane 3) or anti-CPEB1 antibody (lanes 4–5; pc, the polyclonal antibody labeled 'pc' in (A)). Anti-CPEB1 did not immunoprecipitate any cross-linked ^32^P-labeled CPE probe (lane 4). The bands at 70 kDa and 50 kDa on the western blot are most likely non-specific, as they do not correspond to any ^32^P signal. WB, western blot.

To confirm that the CPEB1 MO affected translation of endogenous CPEB1 mRNA, we performed western blots of stage 41 retinas using two antibodies against *Xenopus *CPEB1 (labeled as 'pc' and 'pept' in Figure 4A) and one against human CPEB1 ('2B7'), each directed against different epitopes in CPEB1 (see Materials and methods). Despite the expression of CPEB1 mRNA (Figure 2), CPEB1 protein was not detectable by western blot in embryonic eyes at stage 35/36, 41, and 45, or in stage 45 heads (Figure 4A), although all three antibodies detect *Xenopus *CPEB1 in stage VI oocytes (Figure 4A) and recombinant CPEB1-RBM-GFP expressed in embryos by blastomere injection of CPEB1-RBM-GFP mRNA (Figure 4B). The 2B7 monoclonal anti-CPEB1 antibody detects a band in embryonic tissue 2–3 kDa smaller than the CPEB1 in oocytes, but this is most likely non-specific binding, as neither of the other two antibodies recognizes it. We also tested a commercially available antibody against human CPEB1 that reportedly detects CPEB1 in *Xenopus *larval brains [36]. Using the same western blot methods as that study, we found that this antibody detected multiple non-specific bands and none could be identified as CPEB1 in embryonic eyes and brains up to stage 48 or even in oocytes (data not shown). We also did not detect CPEB1 in stage 48 brains using the two anti-*Xenopus *CPEB1 antibodies ('pc' and 'pept'; data not shown). Although these results do not exclude the possibility of very low levels of CPEB1 protein in the retina (see Discussion), they do call into question what function such a minute amount of CPEB1 could have. Other proteins in the CPEB1 complex, such as Symplekin, Gld2, and PARN, are present in the retina, but seem to be mainly localized to the nucleus in RGCs (Additional file 1).

However, given that developing embryos may use different translational regulators to perform similar functions at different times [53], we hypothesized that other proteins may assume the role of CPEB1 in regulating CPE-containing mRNAs in embryos. Therefore, we asked whether there are other CPE-binding proteins in the retina using UV cross-linking. We incubated stage 41 eye extracts with ^32^P-labeled probes consisting of the 3' untranslated region (UTR) of *Xenopus *cyclin B1, with its CPE motifs intact or mutated. After UV cross-linking, the proteins were resolved by SDS-PAGE; two bands, at approximately 60 kDa and approximately 95 kDa, were bound to the CPE probe but not the mutated probe (Figure 4C). Although CPEB1 is approximately 60 kDa, the 60 kDa CPE-binding protein we detected is most likely not CPEB1, because western blots with anti-CPEB1 antibodies did not detect the 60 kDa ^32^P-labeled band, and because immunoprecipitation with anti-CPEB1 did not precipitate any ^32^P-labeled probe. These results indicate that at least two proteins in the retina bind specifically to CPE sequences.

### Interfering with endogenous CPE-binding proteins impairs axon outgrowth

**Figure 5:**
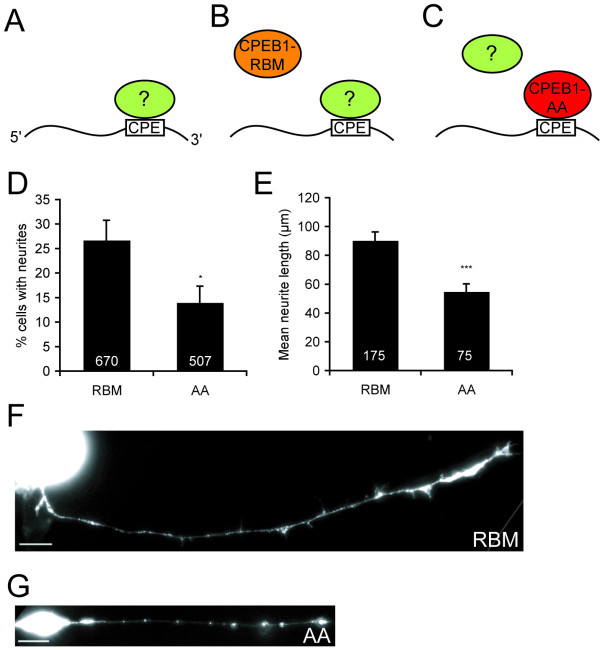
**The CPEB1 mutant (S174A, S180A) (CPEB1-AA) disrupts neurite outgrowth *in vitro***. **(A-C) **Model for how CPEB1-AA competitively interferes with the function of endogenous cytoplasmic polyadenylation element (CPE)-binding proteins. Normally, unidentified CPE-binding proteins regulate the translation or localization of CPE-containing mRNAs (A). The CEPB1-RNA binding mutant (C529A, C539A) (CPEB1-RBM), being unable to bind to CPE-containing RNAs, does not affect this situation and thus serves as a negative control (B). However, CPEB1-AA binds to the CPE, displacing native CPE-binding proteins, thus preventing CPE-mediated mRNA regulation (C). **(D) **Fewer retinal cells transfected with CPEB1-AA-GFP (AA) have neurites compared to cells transfected with CPEB1-RBM-GFP (RBM). **(E) **AA-expressing dissociated retinal neurons have shorter neurites than those expressing RBM. Neurite length was quantified by digitally tracing the longest neurite of each transfected neuron (see Materials and methods for details). **(F, G) **Dissociated retinal neurons transfected with RBM (F), or AA (G). Note the presence of CPEB1-GFP in the neurites. *p < 0.05; ***p < 0.001. Scale bars: 15 μm. Error bars represent standard error of the mean.

Given the presence of CPE-binding proteins in the retina, we addressed the role they might play in retinal axon guidance. Because the short length of the CPE sequence makes it impractical to block CPE-binding using CPE antisense oligonucleotides, we competitively interfered with the function of CPE-binding proteins using CPEB1 mutants. One mutant, CPEB1-AA, has two serine residues (S174 and S180) mutated to alanines, so that it cannot undergo the phosphorylation critical for activating the translation of its target mRNAs in other systems like *Xenopus *oocytes [23]. CPEB1-AA would compete with the endogenous CPE-binding proteins for CPE motifs, and mRNAs with CPEs would be mis-regulated by being bound by CPEB1 rather than their natural CPE-binding proteins (Figure 5C); thus, CPEB1-AA acts as a dominant negative in inhibiting CPE-mediated mRNA regulation. Overexpression of CPEB1-AA prevents oocyte maturation [23] and cerebellar long-term depression and motor learning [35]. For a negative control, we used a CPEB1 mutant defective in RNA binding (CPEB1-RNA binding mutant, or RBM) with point mutations (C529A, C539A) in the zinc-finger domain that abolish its binding to CPE-containing RNAs [54], in order to control for non-specific effects of CPEB1 overexpression unrelated to its RNA binding capability (Figure 5B). GFP was fused to the carboxyl terminus of these constructs [30] to allow visualization of transfected cells and axons, and the constructs were electroporated into the retina at stage 28 [55].

We first asked whether CPEB1-AA-GFP (hereafter AA) would affect retinal axon guidance *in vitro*. Retinal explant cultures from AA-transfected eyes did not yield any GFP-positive axons, even though GFP signal was visible in the explant; this lack of axon outgrowth prevented us from testing whether CPEB1-AA prevents Sema3A-mediated collapse. To test whether AA-transfected RGCs form axons that are too short to exit the explant, we performed dissociated retinal cell culture using eyes with AA or CPEB1-RBM-GFP (hereafter RBM). AA-transfected cells had a reduced rate of neurite formation compared to RBM-transfected cells (Figure 5D), and the neurites that did form were significantly shorter (Figure 5E–G). In preliminary experiments, a similar inhibition of neurite outgrowth was also observed in cells transfected with wild-type CPEB1 (data not shown). These results suggest that disruption of CPE-mediated mRNA regulation by CPEB1-AA causes defects in neurite outgrowth.

**Figure 6:**
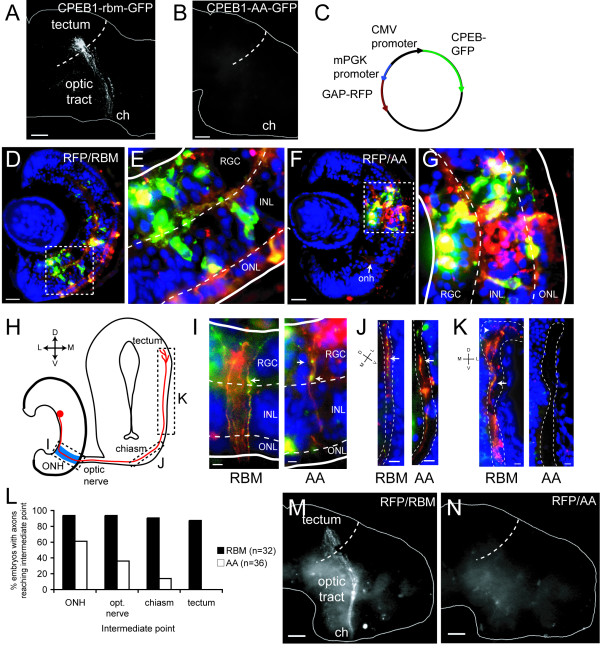
**The CPEB1 mutant (S174A, S180A) (CPEB1-AA) disrupts axon outgrowth *in vivo***. **(A) **Retinal ganglion cells (RGCs) transfected with CPEB1-RBM-GFP (RBM) send axons to the optic tectum correctly. ch, optic chiasm. **(B) **Embryos where RGCs are transfected with CPEB1-AA-GFP (AA) do not have GFP-positive axons in the brain. **(C) **Schematic of bidirectional plasmid encoding both GAP-red fluorescent protein (RFP) and CPEB1-green fluorescent protein (GFP). CMV, cytomegalovirus; mPGK, mouse phosphoglycerate kinase. **(D-G) **Electroporation of bidirectional plasmids causes co-expression of GAP-RFP and CPEB1-GFP. Dashed boxes in (D) and (F) are shown at higher resolution in (E) and (G), respectively. **(H) **Diagram of optic pathway in horizontal sections. Dashed boxes indicate the part of the pathway shown in (I), (J), and (K). **(I) **RFP/RBM- and RFP/AA-transfected axons in the optic nerve head. These axons contain CPEB1-GFP (arrows). **(J) **Very rarely, an RFP/AA-transfected axon extends just beyond the optic chiasm. Dashed lines indicate expected path of retinal axons. **(K) **RFP/RBM-transfected, but not RFP/AA-transfected, axons reach and branch in the tectum (arrowhead). The RFP/RBM image is a composite of two adjacent sections. **(L) **Number of RFP/RBM- and RFP/AA-transfected embryos with detectable RFP-positive axons reaching each intermediate target, analyzed in sections. **(M-N) **RFP/RBM-transfected embryos have bright RFP-positive axons in the optic pathway projecting correctly to the tectum, while RFP/AA-transfected embryos do not. Scale bars: 65 μm (A, B, M, N); 10 μm (D, F, J, K); 5 μm (I). Blue, DAPI; green, CPEB1-GFP; red, GAP-RFP. Dashed lines indicate inner and outer plexiform layers and solid lines indicate the apical and basal limits of the retina. Abbreviations: INL, inner nuclear layer; ONL, outer nuclear layer.

Because dissociated retinal cell cultures contain other cell types aside from RGCs, we sought to extend these findings to RGC axon outgrowth *in vivo *by allowing embryos electroporated at stage 28 to grow to stage 41, at which point RGC axons have normally arrived in the tectum and begun branching. RBM-transfected RGCs extended GFP-positive axons correctly to the contralateral optic tectum (Figure 6A). In contrast, no GFP-positive axons were ever seen in the contralateral brain in AA-transfected embryos (0 of 22 brains; Figure 6B), although expression of the construct in RGCs was confirmed by sectioning the electroporated eye or cutting it in half and mounting it (data not shown). This effect was cell-autonomous because co-electroporation of AA and membrane-tagged red fluorescent protein (GAP-RFP) plasmids yielded many RFP-positive, but no GFP-positive, axons navigating correctly to the tectum (data not shown). However, CPEB1-GFP tends to form large discrete puncta in neurites *in vitro *(Figure 3D) [30] and an axon containing only sparse GFP puncta might be difficult to identify as an axon *in vivo*.

**Figure 7:**
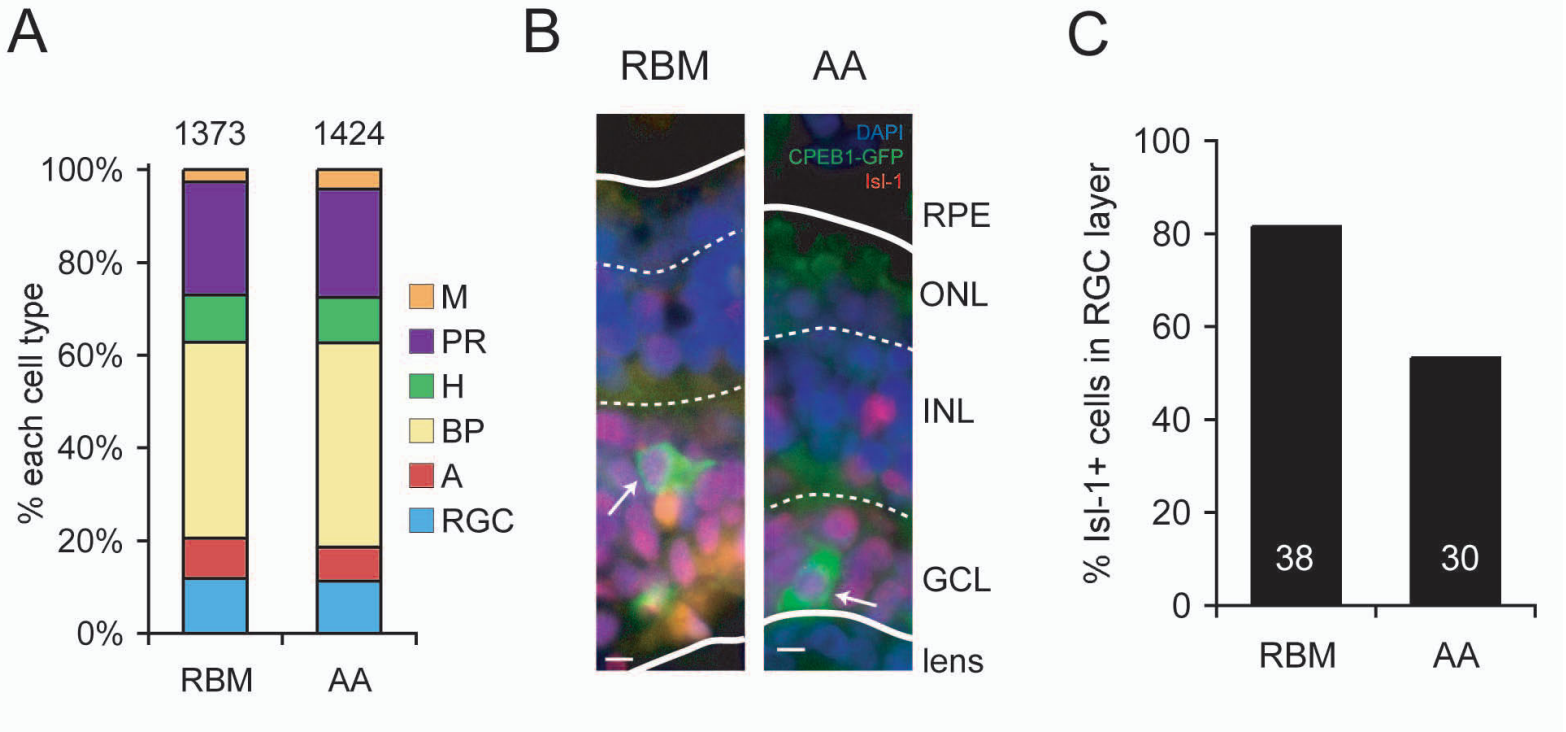
**The CPEB1 mutant (S174A, S180A) (CPEB1-AA) reduces, but does not abolish, retinal ganglion cell (RGC) differentiation**. **(A) **Cells transfected with CPEB1-GFP classified into the six retinal cell types according to layering and morphology; no differences were observed. **(B) **Examples of cells in the RGC layer transfected with CPEB1-RBM-GFP (RBM) or CPEB1-AA-GFP (AA) that are positive for Isl-1 staining. Blue, DAPI; green, GFP; red, Isl-1. In each image, the upper and lower dotted lines indicate the outer and inner plexiform layers, respectively. The upper and lower solid lines indicate the apical and basal limits of the retina, respectively. Numbers in or above bars indicate number of cells counted. **(C) **Fewer cells in the RGC layer transfected with AA express Isl-1 compared to RGCs transfected with RBM. However, approximately half of AA-expressing cells still express Isl-1. Scale bars 5 μm. Abbreviations: A, amacrine cell; BP, bipolar cell; GCL, ganglion cell layer; H, horizontal cell; INL, inner nuclear layer; M, Müller cell; ONL, outernuclear layer; PR, photoreceptor; RPE, retinal pigment epithelium.

We sought to confirm that the lack of axons growing into the brain was not simply due to detection failure by co-transfecting cells with GAP-RFP, ensuring co-expression using a bidirectional plasmid in which CPEB1-GFP is driven by the cytomegalovirus promoter and GAP-RFP is driven by the mouse phosphoglycerate kinase (PGK) promoter (Figure 6C). These constructs were well-expressed in electroporated eyes (Figure 6D–G). Both RBM- and AA-transfected embryos had bright RFP-positive axons in the optic nerve head, where RFP signal colocalized with discrete CPEB1-GFP puncta in AA-transfected embryos and more diffuse CPEB1-GFP in RBM-transfected embryos (Figure 6I). However, in cryosections, such RFP- and GFP-positive axons never extended into the optic tract and only rarely reached the optic chiasm in AA-transfected embryos, whereas they almost always reached the optic tectum in RBM-transfected embryos (Figure 6H–L). In wholemount preparations, RBM-transfected, but not AA-transfected, embryos had bright RFP-positive axons in the contralateral brain (Figure 6M, N). Extremely faint RFP-positive axons could be detected in AA-transfected embryos using a high-sensitivity camera (Additional file 2B), but these never contained CPEB1-GFP and were much dimmer than the RFP-positive axons in the contralateral brain of RBM-transfected embryos (Additional file 2A–C). In contrast, the RFP-positive axons in the optic nerve head and optic nerve often contained CPEB1-GFP and were of similar brightness in RBM- and AA-transfected embryos (Figure 7H; Additional file 2D). Because electroporation introduces random amounts of plasmid into transfected cells, this contrast suggests that of the population of AA-transfected RGCs, only those receiving extremely small amounts of plasmid are able to extend axons into the brain, as they do not express enough CPEB1-AA to have any effect but express just enough GAP-RFP to be visible. Similar defects in axon outgrowth of strongly expressing AA/RFP-transfected neurons were seen after electroporation of the brain by injection into the ventricle (data not shown). Together, these results suggest that while at least some RGCs expressing a substantial amount of CPEB1-AA are able to make axons, they are only short ones.

We next asked whether these effects of CPEB1-AA are specific to axon outgrowth or the result of more general defects in cell survival or differentiation. Using DAPI staining, transfected cells did not have pyknotic nuclei, suggesting that CPEB1 overexpression did not simply cause cell death (Figure 7B). In stage 41 sections of eyes transfected with AA or RBM, cells in the central retina were classified as photoreceptors, horizontal cells, bipolar cells, amacrine cells, RGCs, or Müller cells based on their laminar position and morphology [56,57]. No significant differences were seen (Figure 7A); in particular, for all three conditions, the percentage of cells in the RGC layer was around 11–12%, consistent with the wild-type proportion of RGCs [56].

To test whether CPEB1-transfected cells in the RGC layer differentiate correctly, we determined the relative expression of Islet-1 (Isl-1), a homeodomain transcription factor expressed in RGCs and some bipolar and amacrine cells [58,59] that controls the expression of RGC-specific genes and is required for RGC survival [60,61]. Of cells in the RGC layer, approximately 50% of AA-transfected cells expressed Isl-1, compared to 80% of RBM-transfected cells (Figure 7C). This suggests that CPEB1-AA can affect RGC differentiation, but only for some cells, perhaps depending on the stage in the cell's lifetime when CPEB1 overexpression began. Electroporated constructs are visibly expressed around 6 hours after electroporation [55]; as these embryos were electroporated at stage 28, transgene expression should begin at stage 31. At this stage, future RGCs may range from being in G1 before the final S phase, through having axons nearly reaching the optic chiasm [46,62], creating ample opportunity for variation in the effects of CPEB1 on differentiation. Variability could also stem from the amount of CPEB1 overexpression, but no obvious correlation between GFP expression and Isl-1 expression level was observed. In any case, the finding that 50% of AA-transfected RGCs still express Isl-1 indicates that at least some RGCs differentiate and should be able to send out axons, but their axons rarely reach the optic chiasm and never enter the optic tract, suggesting that at least some of CPEB1-AA's effects are specific to axon outgrowth. Together, these results indicate that CPE-mediated mRNA regulation is required for retinal axon outgrowth.

## Discussion

This study has shown that cytoplasmic polyadenylation is required for growth cone collapse in response to Sema3A, suggesting that cytoplasmic polyadenylation may regulate guidance-cue-induced local translation. The mRNA of a well-known regulator of cytoplasmic polyadenylation, CPEB1, is present at low levels in RGCs, but CPEB1 protein is not detected in the retina, and knockdown of CPEB1 function does not obviously affect retinal axon guidance. However, UV cross-linking revealed the presence of other CPE-binding proteins in the retina, and dominant-negative inhibition of CPE-binding in RGCs causes axon outgrowth defects. These results indicate that cytoplasmic polyadenylation and CPE-mediated regulation of mRNAs are important for RGC axon development.

Cordycepin is a specific inhibitor for mRNA polyadenylation. Although it inhibits RNA synthesis [63], Sema3A-induced growth cone collapse is known to be transcription-independent [2]. Cordycepin can also inhibit deadenylation; an unidentified 3'-5' deadenylase in HeLa cell extracts cannot act on mRNAs capped at the 3' end by a cordycepin residue [64]. However, this is not an entirely off-target effect, as it still addresses cytoplasmic control of poly(A) tail length, and cordycepin could only affect this deadenylase if it is added to the poly(A) tail by polyadenylation. The adenosine control argues against action by cordycepin on adenosine receptors and rules out the possible inhibition of adenylate cyclase by cordycepin, because adenosine and cordycepin inhibit adenylate cyclase activity equally [65]. Finally, our finding that cordycepin slightly reduces but does not abolish the Sema3A-induced increase in translation suggests that cordycepin leaves Sema3A signal transduction pathways relatively intact. Consistent with this, cordycepin does not inhibit *Xenopus *oocyte maturation induced by injection of c-Mos, the synthesis of which occurs early in maturation [40], implying that cordycepin does not affect kinases downstream of c-Mos in oocyte maturation, such as mitogen-activated protein kinase (MAPK) and p90Rsk [66].

We have attempted to detect Sema3A-induced polyadenylation of candidate axonal mRNAs, such as RhoA [11], which contains a CPE in the 3'UTR in *Xenopus *and is polyadenylated in maturing *Xenopus *oocytes (data not shown), but have been hampered by the extremely low amounts of RNA obtainable from isolated *Xenopus *retinal axons, which are not adequate to obtain reliable results with existing PCR-based poly(A) tail assays [67,68]. In addition, it is difficult to say *a priori *which mRNAs might be polyadenylated, as only a handful of mRNAs are known to undergo guidance cue-induced translation in axons [5]. Nevertheless, our cordycepin results imply that axonal mRNAs are polyadenylated in response to Sema3A; future studies using more sensitive, unbiased assays may identify these mRNAs.

The contrast between clear, though weak, detection of CPEB1 mRNA in RGCs and the inability to detect CPEB1 protein in the retina suggests that CPEB1 protein might be present at very low levels. Indeed, in one study, the CPEB1 remaining in oocytes after maturation-associated degradation was undetectable under normal loading and exposure conditions, even though CPEB1 levels in mature oocytes have been variously reported as 25% or 3–5% of CPEB1 levels in immature oocytes [26,69]. Given that CPEB1 was detected in oocytes in this study, we infer from the amount of CPEB1 in oocytes [26] and the amounts of protein loaded in our western blots (see Materials and methods) that the inability to detect CPEB1 in retinas suggests that the relative abundance of CPEB1 protein is about 500 times lower in the retina than in oocytes. This is not implausible, as CPEB1 mRNA levels are also much higher in oocytes than in the embryonic retina (data not shown), and in cDNA libraries collected from *Xenopus tropicalis*, *CPEB1 *is an abundant clone in eggs (0.045% of all clones, compared to 0.023% for ATP synthase, γ-subunit) whereas it is not detected in cDNA libraries between gastrulation and stage 45 [70].

Thus, the inability to detect CPEB1 protein leaves open the possibility that RGCs contain a small amount of CPEB1 protein. However, radiolabeled CPE-containing RNA was bound by proteins that could not be detected by anti-CPEB1 western blot or immunoprecipitation, suggesting that even if a small amount of CPEB1 is present, its function in regulating CPE-containing mRNAs may be taken over by other proteins whose identities are unknown. The other members of the CPEB family, CPEB2–4, are expressed in embryonic eyes (Figure 2F), and although they are not fully cloned in *Xenopus laevis*, human CPEB2 has a predicted molecular weight of 62 kDa, suggesting that the approximately 60 kDa CPE-binding band could be CPEB2. CPEB2–4 have been reported not to bind to one copy of CPE sequence (UUUUAAU) [49], but this may not be true for all mRNAs if the CPE is located in a loop structure. Future work on *Xenopus *CPEB2 awaits cloning of the gene and generation of an anti-XCPEB2 antibody. In addition, KSRP (K-homology splicing regulatory protein; known as VgRBP71 in *Xenopus *and FUBP2, MARTA1, or ZBP2 in other species) binds CPE sequences in mice (Y-SH and JDR, unpublished observations) and regulates the localization of β-actin mRNA in neurons [71]. Although VgRBP71 is 71 kDa [72], its rat homolog MARTA1 (molecular weight 74 kDa) was originally identified as a 90 kDa protein binding to the 3'UTR of microtubule-associated protein 2 mRNA in UV cross-linking assays [73,74], suggesting that the approximately 95 kDa CPE-binding band in Figure 4 could be VgRBP71. Future studies may identify the CPE-binding proteins in the *Xenopus *retina.

The effect of CPEB1-AA suggests that these non-CPEB1 CPE-binding proteins, or at least regulation of CPE-containing mRNAs, are important for RGC axon development. This regulation may occur via mRNA localization, translational repression, translational activation, or all three, as occurs with CPEB1 [18,30]. CPEB1-AA would displace native CPE-binding proteins, thereby causing mis-regulation of CPE-containing mRNAs (Figure 5A–C). Indeed, expression of dominant-negative CPEB1 in Purkinje cells causes defects in cerebellar long-term depression and motor learning, while elimination of endogenous CPEB1 does not [33,35], suggesting that non-CPEB1 CPE-binding proteins are also involved in synaptic plasticity.

Our finding that CPE-mediated mRNA regulation is important for axon outgrowth is consistent with other studies demonstrating roles for post-transcriptional regulation in axon formation and extension. For example, regulation of neurofilament-M mRNA by heterogeneous nuclear ribonucleoprotein (hnRNP) K is required for axon outgrowth in *Xenopus *[75], although hnRNP K is unlikely to be a CPE-binding protein, as it binds to poly(C) sequences [76]. In addition, translational regulation of the neuronal polarity regulator SAD kinase (also known as BR serine/threonine kinase 2 or brain-selective kinase 2) by the mammalian target of rapamycin (mTOR) pathway controls axon formation [77]. It is likely that coordinated regulation of many mRNAs by multiple RNA-binding proteins is required for the complex program of axon extension.

Given that axon extension and growth cone collapse are in some ways opposite phenomena, the effect of dominant-negative CPEB1 on axon extension seems opposed to the requirement for cytoplasmic polyadenylation in growth cone collapse. These can be reconciled by noting that the CPE-binding proteins displaced by CPEB1-AA may not necessarily regulate cytoplasmic polyadenylation, or may regulate the polyadenylation of only a subset of mRNAs that are polyadenylated upon Sema3A stimulation. It would be interesting to directly test the connection between cytoplasmic polyadenylation and the retinal CPE-binding proteins by asking whether cordycepin inhibits axon outgrowth as CPEB1-AA does, or if CPEB1-AA inhibits growth cone collapse as cordycepin does. However, the former experiment would be difficult to interpret given the inhibition of transcription by cordycepin over the timescales required to study neurite outgrowth, while the latter experiment is precluded by the lack of CPEB1-AA-positive axons growing out of transfected retinal explants.

Even if CPE-binding proteins do indeed regulate cytoplasmic polyadenylation, the apparent contradiction described above can be resolved by noting that axon extension and Sema3A-induced collapse occur at different time points of RGC axon development; the effect of CPEB1-AA on axon outgrowth is observed early and most likely includes an effect on neurite initiation (Figure 5D), whereas Sema3A is more effective at collapsing old growth cones (cultured after stage 35/36) than young growth cones [37]. Thus, CPE-mediated mRNA regulation and cytoplasmic polyadenylation may have different roles at different developmental stages. Alternatively, just as protein synthesis is required for both attractive and repulsive responses, CPE-mediated mRNA regulation and cytoplasmic polyadenylation may be involved in both attractive and repulsive responses; future work may examine this possibility.

The conclusion that non-CPEB1 CPE-binding proteins, which may or may not regulate cytoplasmic polyadenylation, are involved in RGC axon outgrowth leaves open the question of how cytoplasmic polyadenylation is regulated. It is not necessarily surprising that different mechanisms would regulate cytoplasmic polyadenylation in oocytes and embryos. For example, even though maternal mRNAs are silenced in immature oocytes from stage I to stage VI, PARN is not expressed until stage III [78,79], suggesting that other mechanisms not involving PARN must deadenylate and silence maternal mRNAs in early immature oocytes. In addition, in early *Drosophila *embryos, regulated translation of germ plasm mRNAs is correlated with their poly(A) tail length, but seems to be independent of the *Drosophila *CPEB homolog ORB [80]. Similarly, in *Xenopus *early embryogenesis, cytoplasmic polyadenylation of mRNAs such as activin receptor is mediated by U-rich sequences (U12–27) similar to, but distinct from, the CPE bound by CPEB1 (U4-6A1-2U) during oocyte maturation [81-83]. These U-rich sequences are bound by ElrA (elav-like ribonucleoprotein A) [84,85], suggesting that ElrA mediates cytoplasmic polyadenylation, although this has not been directly demonstrated. In addition, although ElrA is unlikely to be one of the CPE-binding proteins in Figure 4, as its molecular weight is 36 kDa and it does not bind the cyclin B1 3'UTR, it can bind to the CPE bound by CPEB1 (U4-6A1-2U) in some mRNAs such as cyclin E1 [85]. ElrA is expressed in *Xenopus *throughout development [86], making it a potential regulator of some CPE-containing mRNAs and cytoplasmic polyadenylation in the retina.

In addition to ElrA, a role in regulation of the poly(A) tail length of target mRNAs has been described for other proteins, Musashi [24] and Pumilio [25], as well as the micro-RNA let-7 [87]. Although Musashi (also known as nervous system-specific RNP protein, or Nrp-1) is not expressed in *Xenopus *differentiated RGCs [88], we have detected Pumilio and miRNAs in RGCs (F van Horck and M-L Baudet, unpublished observations). Pumilio and let-7 repress target mRNAs by stimulating deadenylation, as CPEB1 does in immature oocytes. If these or other factors repress and deadenylate mRNAs in unstimulated growth cones, Sema3A stimulation might cause them to release their target mRNAs, allowing them to be polyadenylated by default, which would explain why cordycepin prevents Sema3A-induced collapse. Future studies may determine whether these RNA-binding proteins, micro-RNAs, CPE-binding proteins, or other mechanisms regulate cytoplasmic polyadenylation in RGC axons, aided by the identification and 3'UTR sequence analysis of mRNAs that are polyadenylated upon guidance cue stimulation.

## Conclusion

Our results show for the first time that cytoplasmic polyadenylation is required for growth cone chemotropic responses. We have also shown that regulation of CPE-containing mRNAs is involved in retinal axon outgrowth. These results pave the way for future studies to investigate the identity of the CPE-binding proteins in the *Xenopus *retina and how cytoplasmic polyadenylation is regulated in retinal axons.

## Materials and methods

### Embryos

*Xenopus laevis *embryos were obtained by *in vitro *fertilization, raised in 0.1× Modified Barth's Saline at 14–20°C and staged according to [89].

### Cell culture

Eyes were dissected from stage 37/38 embryos and cultured at 20°C for 24 h in 60% L-15 (Invitrogen; Paisley, UK) on coverslips coated with 10 μg/ml poly-L-lysine and 10 μg/ml laminin (Sigma; Gillingham, UK). For dissociated culture, eyes were electroporated at stage 26–28 and dissected at stage 32–33, cut in half, trypsinized for 2 minutes, dissociated in Ca^2+^-free medium with 1% bovine serum albumin, and plated on coverslips coated with 100 μg/ml poly-L-lysine and 10 μg/ml laminin in 60% L-15 with 5% fetal bovine serum (Gibco). GFP-positive cells were imaged at 100× on a Nikon TE2000-U microscope. Cells with and without neurites were counted and for those with neurites, the length of the longest neurite was measured by tracing in Open*lab *(Improvision; Coventry, UK) using a digital pen tablet (Wacom). A6 kidney epithelial cells were thawed from a lab stock and grown in 60% L15 medium with penicillin-streptomycin-fungizone (PSF) and 5% serum.

### Collapse assays

Stage 37/38 retinal explant cultures were pre-incubated for 30 minutes with 200 μM cordycepin or 200 μM adenosine (Sigma), then treated with the collapsing agent (2 μg/ml Sema3A-Fc (R&D Systems; Minneapolis, MN), control medium, 1 μM lysophosphatidic acid (Sigma)) for 10 minutes and fixed. For experiments on severed axons, cordycepin or adenosine was applied immediately after severing. Collapsed and non-collapsed growth cones were counted in a blinded manner. A growth cone was counted as collapsed if it had no lamellipodia and two or fewer filopodia, each shorter than 10 μm.

### Measurement of protein synthesis levels

^3^H-leucine incorporation assays were performed as described [2]. For puromycin labeling, retinal cultures were treated with cordycepin, adenosine, puromycin (Sigma), LnLL (Sigma), anisomycin (Sigma), and/or Sema3A-Fc as described in Figure 2F. After treatment, the entire solution was removed and the cultures were lysed in 50 μl RIPA buffer and subjected to SDS-PAGE and western blotting with anti-puromycin antibody.

### Electroporation

Electroporation was performed as described [55]. Briefly, anesthetized stage 28 embryos were placed in the longitudinal channel of a t-shaped Sylgard chamber and flat-ended 0.5 mm wide platinum electrodes (Sigma) were placed in the ends of the transverse channels. Glass capillaries were filled with carboxyfluorescein-tagged antisense MO (1 mM) or DNA (1–4 μg/μl in water). Approximately 10–30 nl of MO or DNA solution was injected into the space between the eye and the brain. Injections stopped and the capillary was removed immediately before the first electric pulse was delivered by the square wave pulse generator (TSS20 OVODYNE electroporator, Intracel; Royston, UK). The pulse series consisted of 8 pulses, 18–20 V, 25–50 ms long, 1s apart.

### Imaging and analysis of transfected embryos

Embryos were fixed in 4% formaldehyde for 1–2 h at room temperature. For wholemount preparations, the brain was dissected out and split in half along the midline to exclude brains with extra-retinal transfection. The two half-brains were mounted lateral side up. For sections, 8–25 μm horizontal cryosections were cut from embryos equilibrated in 30% sucrose and embedded in Tissue-Tek O.C.T. compound (Sakura Finetek; Zoeterwoude, the Netherlands). Wholemounted brains and sections were imaged at 20× and 40× on a Nikon Eclipse 80i upright microscope, using constant video settings for quantitative analysis of axon brightness. In cases where axons did not lie in a single focal plane, a z-stack was taken and a composite image was created using Open*lab*. The brightest retinal axon in each sample was digitally traced in ImageJ, and the average intensity along the axon was measured. The background intensity to be subtracted from this value was taken as the average intensity along a freehand line drawn along both sides of the axon of interest, as close as possible to the axon in an area free of other labeled axons.

### Plasmids

GAP-RFP was subcloned by PCR using the primers 5'-GAAGATCTATGCTGTGCTGTATGAGAAG-3' and 5'-CGGCTAGCCTAGGCGCCGGTGGAGTGGC-3', digested with *Nhe*I and *Bgl*II, and ligated into *Nhe*I/*Bgl*II-digested mPGK-biCS2+ (gift of D Turner, University of Michigan). To create the CPEB1(S174A, S180A)-GFP (CPEB1-AA-GFP) fusion construct, the stop codon of full-length CPEB1 was mutated to a cysteine by PCR amplification; the PCR product was digested with *Hin*dIII and *Bgl*II and the 370 bp DNA fragment was cloned into *Hin*dIII/*Bam*HI-digested pEGFP-N1 (Clontech; Mountain View, CA). The resulting plasmid was digested with *Bgl*II and *Hin*dIII and ligated with the 1.3 kb *Bgl*II and *Hin*dIII fragment of CPEB1-AA excised from myc-CPEB1-AA [23]. CPEB1(C529A, C539A)-GFP (CPEB1-RBM-GFP) was created previously [30]. CPEB1-AA-GFP and CPEB1-RBM-GFP were subcloned by PCR using the primers 5'-CGGAATTCATGGCCTTCCCACTGAAAGA-3' and 5'-GCTCTAGATTACTTGTACAGCTCGTCCA-3' followed by *Eco*RI/*Xba*I digestion and ligation into *Eco*RI/*Xba*I-digested pCS2+ or mPGK-biCS2+-GAP-RFP. Gld2(D242A)-GFP was created by replacing CPEB1-RBM with Gld2(D242A) in pCS2+-CPEB1-RBM-GFP. Gld2(D242A) was amplified by PCR from pMyc-Gld2(D242A) [22] using the primers 5'-CGGAATTCATGTACCCTAACTCCCCCAG-3' and 5'-CGACCGGTCCTAACGAGTGCATTTTTTTC-3', digested with *Eco*RI and *Age*I, and ligated into *Eco*RI/*Age*I-digested pCS2+-CPEB1-RBM-GFP.

### Wholemount *in situ *hybridization

Sense and antisense Dig-labeled riboprobes were transcribed *in vitro *from the full-length sequence of *Xenopus *CPEB1 in pBluescript. After quantification of Dig-incorporation to match sense and antisense probe concentrations, wholemount *in situ *hybridization was carried out as described [90].

### Blastomere injection

Blastomere injection of MOs (10 ng/cell at two-cell stage) and mRNA (1 ng) transcribed *in vitro *using the mMESSAGE mMACHINE kit (Ambion; Austin, TX) was performed as described [91].

### Laser capture microdissection

Stage 41 embryos were lightly fixed (4% formaldehyde, 5–10 minutes) and 8 μm horizontal cryosections were collected on a PEN-membrane slide (Leica Microsystems; Wetzlar, Germany). The RGC layer was microdissected out of these sections using a Leica LMD6000 laser microdissection system and collected in 20 μl lysis buffer.

### RT-PCR

RNA was extracted using Qiagen RNeasy kits and RT-PCR was performed using theOneStep RT-PCR kit (Qiagen; Crawley, UK). Primers were as shown in Table 1.

**Table 1:** Primers used for RT-PCR

Name	Sequence (5'-3')	GenBank accession number of gene
xGld2 f	TGACCACATAGACACCACTTTGCC	AY655140
xGld2 r	CGCACCACTGACTTTATCCCTG	
Symplekin f	GCTATCTCCAGCATCAACCTTACG	BC047265
Symplekin r	TGTCCCCCTTCACCATCTTCTC	
PARN f	AACACATCCCTTGCCGAACTG	14495248
PARN r	GGTAGAGGTCACTGGTCTTCCATTC	
CPEB1 f	GCAACTTTGTGCGTAGTCCA	NM_001090603
CPEB1 r	TCCATAGAGTGCTGCCAGTG	
CPEB2 f	CCATCAAAGCAGTGGTTGGAAC	BX850448
CPEB2 r	GAACGAGTGAACTTGGGTGGTG	
CPEB3 f	CAGTCAGTTTGTGGTAAGCAGTCG	BJ617668
CPEB3 r	ATGGGGACAGAGATGGGGTG	
CPEB4 f	CAAAGTCCATCACCAACACCCTC	CF547305
CPEB4 r	CCATCATCCAGAAATCCATCTTCC	
β-actin f	CCTGTGCAGGAAGATCACAT	BC041203
β-actin r	TGTTAAAGAGAATGAGCCCC	

### DiI filling

DiI filling was performed essentially as described [92,93]. E17–E19 CPEB1 +/- and CPEB1 -/- mouse embryos were fixed in 4% formaldehyde. Small crystals of 1,1'-dioactadecyl-3, 3, 3', 3'-tetramethylindocarbocyanin perchlorate (DiI; Invitrogen) were inserted into the optic disc using fine forceps. Embryos were incubated in 4% formaldehyde for 6–10 weeks at 32°C. Labeled brains were imaged on a Leica MZFLIII epifluorescence microscope. For *Xenopus *embryos, a solution of DiI crystals dissolved in chloroform (Sigma) was loaded into a glass capillary. The lens was removed and the DiI solution was injected into the eye, ensuring that the DiI droplet that formed contacted the optic fiber layer. Embryos were incubated at room temperature for 2 days before dissection.

### Immunofluorescence

#### Wholemount

Fixed mouse retinas were dissected from E17–E19 wild-type, CPEB1 +/- and CPEB1 -/- embryos, and the lenses were removed. Retinas were washed 3 × 10 minutes and 1 × 30 minutes in PBT (1× phosphate-buffered saline (PBS), 0.2% bovine serum albumin, 0.5% Triton), blocked for 60 minutes in PBT + 10% heat-inactivated goat serum, incubated in primary antibody in blocking buffer overnight at 4°C, washed for 2 × 10 minutes and 3 × 30 minutes in PBT, incubated in Cy3-conjugated anti-mouse antibody in blocking buffer for 1 h, washed 5 × 20 minutes in PBT, and flattened and mounted.

#### Sections

Sections were air-dried and OCT was removed by 2 × 5 minutes washes in 1× PBS. For Isl-1 staining, slides were pre-treated with 0.01 M sodium citrate, pH 6.0 at 95°C for 10 minutes to expose the Isl-1 epitope. Slides were washed 3 × 5 minutes in PBT (1× PBS + 0.01% Triton X-100), blocked 20 minutes in PBT + 10% HIGS, incubated with primary antibody for 1 h, washed 3 × 5 minutes with PBT, incubated with secondary antibody for 45 minutes, followed by DAPI (4',6-diamidino-2-phenylindole; 50 ng/ml in PBT) for 10 minutes and 3 × 5 minute washes with PBT, and mounted in FluoroSave. fluorescein isothiocyanate (FITC)-conjugated goat anti-GFP (1:500; Abcam; Cambridge, UK) was used on heated slides to recover GFP signal.

#### Western blots

Samples were lysed in RIPA buffer (Sigma) with a protease inhibitor cocktail (Sigma) on ice for 30 minutes, homogenized and centrifuged, and the supernatant was taken and boiled in sample buffer for 5 minutes. The lysate of approximately 10 eyes or 0.5 oocytes was loaded on each lane. Samples were run through a 4% stacking gel at 50 V and an 8–12% resolving polyacrylamide gel at 50–150 V, then transferred onto a nitrocellulose membrane at 4°C and 40 mA overnight. Membranes were blocked for 1–2 h in TBST (5 mM Tris pH 8.0, 150 mM NaCl, 0.05% Tween-20) with 5% dry milk, incubated in primary antibody for 1–2 h at room temperature or overnight at 4°C in TBST with 0.5% milk, washed twice in TBST without milk for 15 minutes each, incubated in secondary antibody conjugated to horseradish peroxidase (HRP) in TBST with 0.5% milk for 45 minutes at room temperature, and washed 3 times in TBST for 15 minutes each. HRP was detected with ECL Plus (GE Healthcare; Chalfont St. Giles, UK) and X-ray film (Kodak).

### UV cross-linking and immunoprecipitation

UV cross-linking was performed as described [54]. Briefly, the 3'UTR of *Xenopus *cyclin B1 mRNA containing or lacking two CPE sequences [94] was transcribed *in vitro *with ^32^P-UTP and purified on a DyeEx column. Stage 41 eyes were lysed in immunoprecipitation buffer (25 mM HEPES pH 7.5, 150 mM NaCl, 10% glycerol, 1 mM DTT, 2 mM EDTA, 1 mM MgCl_2_, 0.5% Triton X-100, 2 mM sodium orthovanadate, 2 mM beta-glycerophosphate, and protease inhibitor cocktail (Roche Applied Science; Indianapolis, IN). Eye lysate was incubated with the radiolabeled RNA probe (2.6 × 10^5 ^cpm) for 10 minutes on ice and 10 minutes at room temperature followed by UV cross-linking (440 mJ; UV Stratalinker 1800) and RNase A digestion of unprotected RNA. Cross-linked samples were pre-cleared with IgG-conjugated sepharose beads and incubated with 2 μl rabbit IgG or anti-CPEB1 antibody and 30 μl protein A-sepharose beads overnight at 4°C, and washed 5 times in immunoprecipitation buffer (0.05% Triton instead of 0.5%). Input and immunoprecipitated samples were boiled in sample buffer, run on 10% SDS-PAGE, transferred to a PVDF membrane, and autoradiographed. The membrane was then subjected to western blot with anti-CPEB1.

### Reagents and antibodies

Cordycepin and adenosine were dissolved in culture medium to a stock concentration of 4 mM and stored at -20°C. Recombinant human Sema3A-Fc chimera (R&D Systems) was reconstituted in 0.1% protease-free bovine serum albumin (Sigma) in 1× PBS, aliquoted, and stored at -80°C. The carboxyfluorescein-tagged anti-CPEB1 MO was 5'-ATCATCTTTCAGTGGGAAGGCCATG-3' (Gene Tools; Philomath, OR). Anti-CPEB1 antibodies were: the 2B7 mouse monoclonal antibody against human CPEB1, 1:1000 ('2B7') [95]; a rabbit antibody against two *Xenopus *CPEB1 peptides, 1:12,000 ('peptide') [69]; and a rabbit antibody against the amino terminus of *Xenopus *CPEB1 ('pc'). Other primary antibodies were: mouse anti-β-tubulin, 1:100 (E7, DSHB; Iowa City, IA); mouse anti-Isl-1, 1:100 (39.4D5, DSHB); mouse anti-myc, 1:100 (9E10, DSHB); FITC-tagged goat anti-GFP for immunofluorescence, 1:500 (Abcam); mouse anti-GFP for western blotting, 1:1,000 (Roche); rabbit anti-puromycin, 1:500 (custom-made antiserum from Eurogentec; Fawley, UK); mouse anti-β-actin, 1:1000 (AC15, Abcam); mouse anti-Symplekin, 1:200 (immunofluorescence) and 1:2000 (western blot) (BD Biosciences; Franklin Lakes, NJ); rabbit anti-PARN, 1:2,000 (gift of M Wormington); rabbit anti-Gld2, 1:500 [19]. Secondary antibodies were: Alexa 594-conjugated donkey anti-rabbit, 1:1,500 (Molecular Probes); Cy3-conjugated goat anti-mouse 1:700 (Calbiochem; San Diego, CA); HRP-conjugated goat anti-mouse, 1:5,000–10,000 (Abcam); HRP-conjugated goat anti-rabbit 1:20,000 (Zymed).

## Abbreviations

AA: CPEB1-AA-GFP; CPE: cytoplasmic polyadenylation element [consensus (U)4–5(A)1-2U]; CPEB: CPE-binding protein; CPEB1-AA: CPEB1 mutant (S174A, S180A); CPEB1-RBM: CEPB1-RNA binding mutant (C529A, C539A); DAPI: 4',6-diamidino-2-phenylindole; E: embryonic day; FITC: fluoroescein isothiocyanate; GFP: green fluorescent protein; Gld2: Germ-line development factor 2; Isl-1: Islet-1; MO: morpholino; PARN: poly(A) ribonuclease; PBS: phosphate-buffered saline; PGK: phosphoglycerate kinase; RBM: CPEB1-RBM-GFP; RFP: red fluorescent protein; RGC: retinal ganglion cell; Sema3A: Semaphorin3A; UTR: untranslated region.

## Competing interests

The authors declare that they have no competing interests.

## Authors' contributions

ACL, JDR, and CEH designed the experiments. ACL performed and analyzed most of the experiments and wrote the manuscript. CLT performed the collapse assays in Figure 1A–E and 3C, F. CLL performed the UV cross-linking experiments in Figure 4C. LS developed the puromycin labeling technique. YSH created the CPEB1-AA-GFP construct.

## Supplementary Material

Additional file 1 **Gld2, PARN, and Symplekin are expressed in the retina, mainly in the nucleus**. **(A, B) **Gld2, PARN, and Symplekin are detected in *Xenopus *embryonic eyes by RT-PCR (A)and western blot (B). **(C-E) **Sections of stage 41 eyes electroporated with Gld2(D242A)-GFP. Gld2(D242A)-GFP is mainly localized in the nucleus (arrows) but can sometimes be cytoplasmic in photoreceptors (D, arrowhead), with some faint cytoplasmic signal also seen in some RGCs (E, arrowhead). Green, Gld2-GFP; blue, DAPI. **(F, G) **Sections of stage 41 eyes electroporated with myc-PARN(D28A), which is also localized to the nucleus in most cells (arrows), with occasional cytoplasmic localization in photoreceptors (arrowhead). Red, myc-PARN; blue, DAPI. **(H-J) **Immunohistochemistry on sections of stage 41 *Xenopus *eye with anti-Symplekin antibody reveals nuclear localization of endogenous Symplekin. (H) DAPI, (I) Symplekin, (J) merge. Note that Symplekin is not expressed at the ciliary margin (arrows). Red, Symplekin; blue, DAPI. Scale bars: 10 μm (C-E); 5 μm (F, G); 30 μm (J). In (C-E), the upper and lower dashed lines indicate the outer and inner plexiform layers, respectively, while the upper and lower solid lines indicate the retinal pigment epithelium and optic fiber layer, respectively. Click here for file

Additional file 2 **Extremely faint RFP-positive axons can be detected in the optic pathway of GAP-RFP/CPEB1-AA-GFP-transfected embryos**. **(A) **Diagram of optic pathway in wholemount brains. Dashed box indicates the area shown in higher magnification in (B). **(B) **RFP-positive axons are much brighter in GAP-RFP/CPEB1-RBM-GFP-transfected embryos than in GAP-RFP/CPEB1-AA-GFP-transfected embryos. These are the brains shown in Figure 3J, K imaged with more sensitive camera settings. These images were captured under identical video settings and displayed with identical contrast enhancement. **(C) **Quantification of axon intensity in the optic tract. **(D) **RFP-positive axons in the optic nerve head (ONH) have similar intensity in RFP/RBM- and RFP/AA-transfected embryos (Figure 3H). **p < 0.01. Scale bars: 30 μm. Error bars represent standard error of the mean. Click here for file
