# Supplementary figures and images for: Cytoplasmic polyadenylation and cytoplasmic polyadenylation element-dependent mRNA regulation are involved in Xenopus retinal axon development
Source: Neural Dev. 2009 Mar 2;4:8. doi: 10.1186/1749-8104-4-8 (PMC2661069; doi:10.1186/1749-8104-4-8)

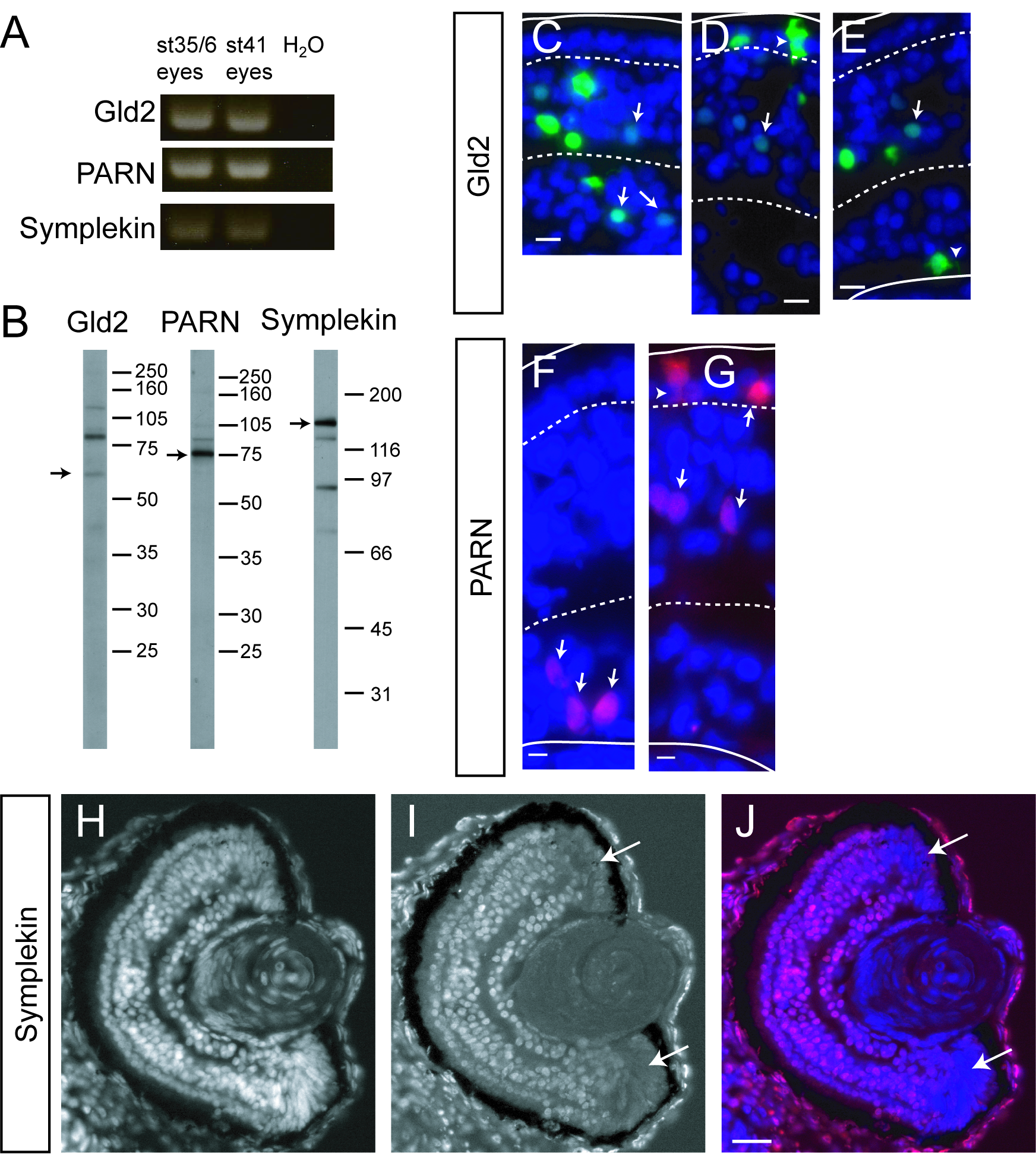

Supplement: Additional file 1 — Gld2, PARN, and Symplekin are expressed in the retina, mainly in the nucleus. (A, B) Gld2, PARN, and Symplekin are detected in Xenopus embryonic eyes by RT-PCR (A)and western blot (B). (C-E) Sections of stage 41 eyes electroporated with Gld2(D242A)-GFP. Gld2(D242A)-GFP is mainly localized in the nucleus (arrows) but can sometimes be cytoplasmic in photoreceptors (D, arrowhead), with some faint cytoplasmic signal also seen in some RGCs (E, arrowhead). Green, Gld2-GFP; blue, DAPI. (F, G) Sections of stage 41 eyes electroporated with myc-PARN(D28A), which is also localized to the nucleus in most cells (arrows), with occasional cytoplasmic localization in photoreceptors (arrowhead). Red, myc-PARN; blue, DAPI. (H-J) Immunohistochemistry on sections of stage 41 Xenopus eye with anti-Symplekin antibody reveals nuclear localization of endogenous Symplekin. (H) DAPI, (I) Symplekin, (J) merge. Note that Symplekin is not expressed at the ciliary margin (arrows). Red, Symplekin; blue, DAPI. Scale bars: 10 μm (C-E); 5 μm (F, G); 30 μm (J). In (C-E), the upper and lower dashed lines indicate the outer and inner plexiform layers, respectively, while the upper and lower solid lines indicate the retinal pigment epithelium and optic fiber layer, respectively. [file 1749-8104-4-8-S1.tiff]

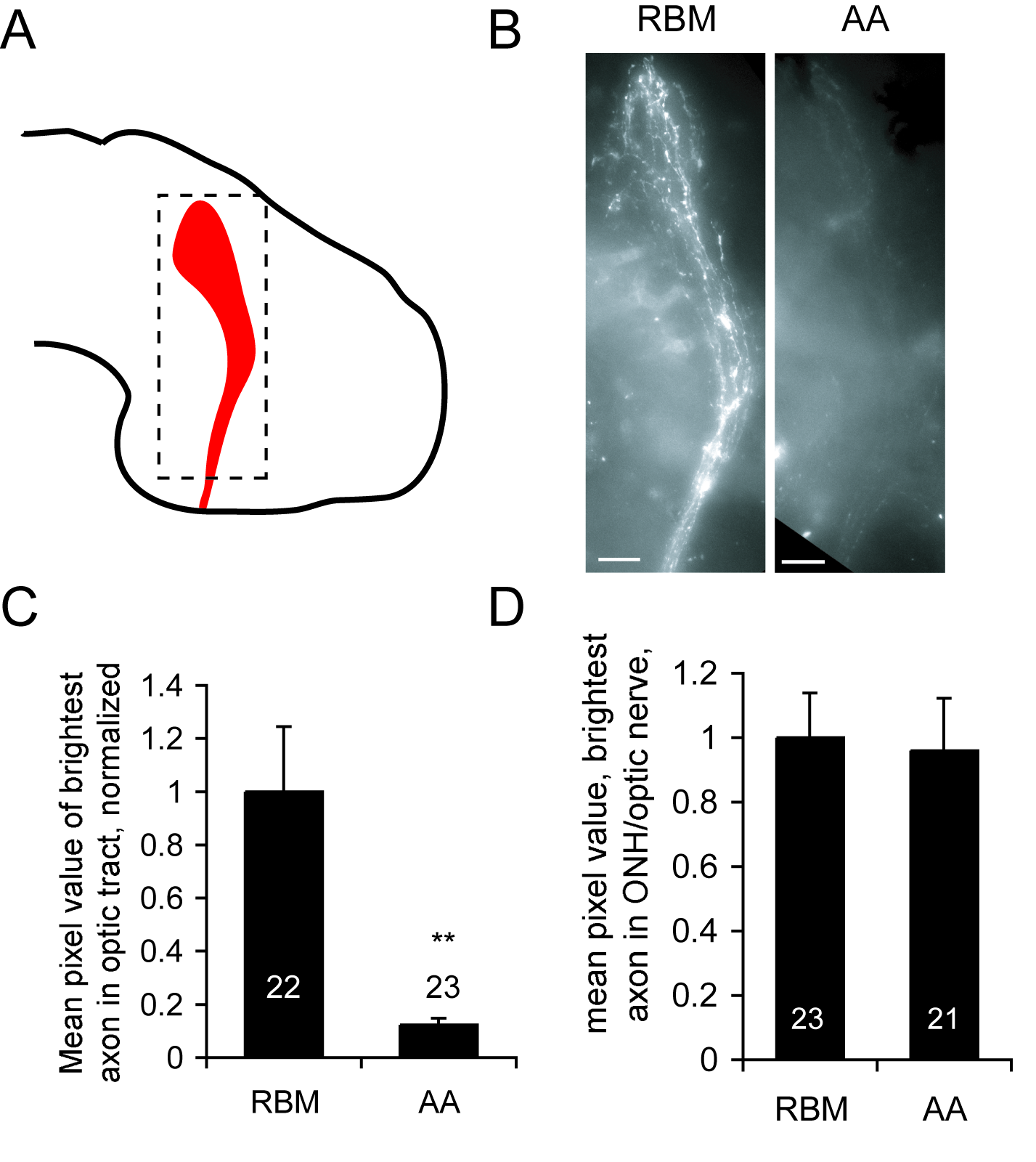

Supplement: Additional file 2 — Extremely faint RFP-positive axons can be detected in the optic pathway of GAP-RFP/CPEB1-AA-GFP-transfected embryos. (A) Diagram of optic pathway in wholemount brains. Dashed box indicates the area shown in higher magnification in (B). (B) RFP-positive axons are much brighter in GAP-RFP/CPEB1-RBM-GFP-transfected embryos than in GAP-RFP/CPEB1-AA-GFP-transfected embryos. These are the brains shown in Figure 3J, K imaged with more sensitive camera settings. These images were captured under identical video settings and displayed with identical contrast enhancement. (C) Quantification of axon intensity in the optic tract. (D) RFP-positive axons in the optic nerve head (ONH) have similar intensity in RFP/RBM- and RFP/AA-transfected embryos (Figure 3H). **p < 0.01. Scale bars: 30 μm. Error bars represent standard error of the mean. [file 1749-8104-4-8-S2.tiff]
